# Improved laccase production by *Trametes versicolor* using Copper-Glycyl-L-Histidyl-L-Lysine as a novel and high-efficient inducer

**DOI:** 10.3389/fbioe.2023.1176352

**Published:** 2023-04-25

**Authors:** Feng Wang, Xiaolei Yu, Zhuo Yu, Yi Cui, Ling Xu, Shuhao Huo, Zhongyang Ding, Liting Zhao, Lizhi Du, Yanguo Qiu

**Affiliations:** ^1^ School of Food and Biological Engineering, Jiangsu University, Zhenjiang, China; ^2^ Institute of Agricultural Products Processing Engineering, Jiangsu University, Zhenjiang, China; ^3^ Laboratory of Carbohydrate Chemistry and Biotechnology, Ministry of Education, School of Biotechnology, Jiangnan University, Wuxi, China; ^4^ Shandong Dehemingxing Biotechnology Co., Ltd., Weifang, China

**Keywords:** *Trametes versicolor*, laccase, copper-Glycyl-L-Histidyl-L-Lysine, medium optimization, induction

## Abstract

A highly efficient strategy using Copper-Glycyl-L-Histidyl-L-Lysine (GHK-Cu) as a novel inducer was developed to enhance laccase production by *Trametes versicolor*. After medium optimization, laccase activity increased by 12.77-fold compared to that without GHK-Cu. The laccase production of 1113.8 U L^−1^ was obtained by scaling-up culture in 5-L stirring tank. The laccase production induced by CuSO_4_ was poorer than that of GHK-Cu at the same mole concentration. GHK-Cu could increase the permeability of cell membrane with less damage, and it facilitated the adsorption, accumulation, and utilization of copper by fungal cells, which was beneficial for laccase synthesis. GHK-Cu induced better expression of laccase related genes than that of CuSO_4_, resulting in higher laccase production. This study provided a useful method for induced production of laccase by applying GHK chelated metal ion as a non-toxic inducer, which reduced the safety risk of laccase broth and provided the potential application of crude laccase in food industry. In addition, GHK can be used as the carrier of different metal ions to enhance the production of other metalloenzymes.

## 1 Introduction

Laccase (phenol-oxygen oxidoreductase; EC 1.10.3.2) is a kind of copper containing polyphenol oxidase. Laccase was firstly isolated and purified from lacquer trees of Southeast Asia in 1894 and subsequently it has been identified in fungi, bacteria, and insects (Khatami et al., 2022). Among them, white-rot fungi are good laccase producers (Pisacha et al., 2020). Laccase has a wide range of substrate specificity, and during catalytic reactions, the substrate can be reduced to water and other small molecule substances without causing secondary pollution. This is crucial for environmental maintenance and applications in various industries. So, as an ideal green biocatalyst, laccase has a wide range of applications, including food, textiles, cosmetics, pharma, biofuels, pulp and paper, and bioremediation (Gomez-Fernandez et al., 2020). The application of laccase in food industry was a hot spot in recent years. However, the large-scale application of laccase in the industry has been limited by the low production and expression levels of natural laccase and the high cost (Su et al., 2020). To improve the laccase expression level, different techniques and methods have been studied, including strain screening, inducer selection, recombinant expression, optimization of medium composition and culture conditions, protein rational design and site-directed mutation (Pardo and Camarero, 2015; Valle et al., 2015; Wang et al., 2017; Wang et al., 2020; Zhang et al., 2021). In addition, new sustainable strategys were developed to improve laccase production, such as physical treatment, and co-culture with other fungi (Wang et al., 2013; Zhang et al., 2020). High enzyme production, high enzyme quality, and excellent performance are the keys to the industrial application of laccase, however, these methods are still far from the industrial wide application (Bertrand et al., 2017). Therefore, it is still attracting the interest of researchers to explore more strategy to improve laccase production.

The most used laccases in the industry were produced by fungi. *T. versicolor* has been recognized as one of the most effective white-rot basidiomycetes to produce large amount of laccase (Fonseca et al., 2016). At present, laccase production of *T. versicolor* has been widely investigated. To improve the activity of laccase, the culture condition of *T. versicolor* was optimized by the orthogonal test in order to improve the activity of laccase (Liu et al., 2019). Researchers found that using cheap substrates to increase laccase production of *T. versicolor* was also an effective strategy, such as tea residues (Xu et al., 2020). In addition, fermentation medium aeration was applied to enhance the laccase production of *T. versicolor* in three types of bioreactors due to the sufficient oxygen supply for microorganisms (Pinheiro et al., 2020).

The selection of inducers in laccase production is an important and interesting work. At present, the reported laccase inducers mainly include phenolic compounds, agro-industrial wastes, natural inducers, aromatic compounds, alcohols, detergents, and metal ions (Wang et al., 2019). Phenolic compounds were often considered effective inducers of laccase, such as guaiacol (Chaurse and Sahay, 2023). The binding of phenolic compounds present in potatoes to starch was believed to be involved in the induced synthesis of laccase in *Pleurotus florida* (Das et al., 1999). Agro-industrial wastes and natural inducers are very economical, and they come from a wide range of sources and provide nutrients (Bertrand et al., 2017). High concentrations of aromatic compounds are toxic to organisms, inhibiting cell growth and enzyme production (BİRhanli and Yesilada, 2017). Alcohols are cheaper, more readily available, and less toxic. However, laccase activity did not increase significantly (Valle et al., 2015). Recent research has shown that the presence of Pb^2+^ could burst the activity of laccase from *Truncatella angustata* BPF5 (Chaurse and Sahay, 2023). More and more studies have proved that metal ions can promote the production of laccase, and Cu^2+^ is more effective than the other metal inducers. However, high concentration of Cu^2+^ accumulated in cells was toxic to cells (BİRhanli and Yesilada, 2017). Therefore, the separation and purification operations were generally applied to remove the residual Cu^2+^ residues from culture broth, which increased the production cost and limited its application in food industry.

Copper-Glycyl-L-Histidyl-L-Lysine (GHK-Cu) is a compound formed by the combination of GHK with Cu^2+^. GHK is non-toxic and occurs naturally in saliva, blood, and urine (Li et al., 2016). Then GHK readily binds copper or zinc cations and was considered as the transporter of metal ions through membranes, which reduced the damage to cells resulted from metal ions (Alshammari and Platts, 2020). In addition, GHK-Cu has antioxidant and anti-inflammatory effects, which can improve the skin and has a wide range of applications in the cosmetics and the skin tissue remodeling industry (Pickart and Margolin, 2018). Therefore, GHK-Cu may be a potential inducer candidate for laccase production from fungi. In this study, laccase production from *T. versicolor* was induced by using GHK-Cu instead of Cu^2+^. For this purpose, the composition of the culture medium was optimized by one-factor-at-a-time and Box-Behnken design (BBD). And the interaction between medium compositions was analyzed by response surface methodology (RSM). The laccase production in the optimal medium was scaling up in a 5 L stirring reactor. The cell membrane permeability, consumption of copper source, and laccase gene expression were investigated using GHK-Cu and were compared to those under Cu^2+^ induction.

## 2 Materials and methods

### 2.1 Materials

#### 2.1.1 Microorganism and chemicals

*T. versicolor* CICC 14001 was purchased from China Strain Preservation Center and was stored on potato dextrose agar slant at 4°C. TaKaRa MiniBEST Plant RNA Extraction Kit, PrimeScript™ RT reagent Kit with gDNA Eraser (Perfect Real Time) Kit, Green^®^ Premix Ex Taq™ II (Tli RNaseH Plus) Kit was purchased from Shanghai Baisai Biotechnology Co., Ltd. All other chemicals were purchased from Sinopharm Chemical Reagent Co., Ltd.

#### 2.1.2 Medium and culture conditions

The seed culture of *T. versicolor* was prepared according to the procedure described by Xu et al. (2020). The seed pellet mycelia of 5 mL were inoculated into the fermentation medium for laccase production. The pH of the culture broth for laccase production was adjusted with 0.1 mol L^-1^ NaOH to 4.4 during the fermentation process until the pH rises automatically. The basic fermentation medium was the following composition (per liter): glucose 2 g, KH_2_PO_4_ 0.2 g, FeSO_4_ ·7H_2_O 0.035 g, MgSO_4_·7H_2_O 0.05 g, NH_4_Cl 0.5 g, CaCl_2_ 0.0755 g and sterilized at 121°C for 20 min. The cultures are incubated at 27°C, 150 rpm. One-factor-at-a-time, Box-Behnken design (BBD) and response surface methodology (RSM) were used to optimize the medium of laccase fermentation induced by GHK-Cu (Supplementary Materials and Methods S1). The optimal culture medium were obtained by sampling from the fermentation broth and measuring laccase activity on the 7th day. The scale-up of the reactor culture and exploration of possible mechanisms both were carried out under the optimal medium conditions. This experiment was conducted in a 5 L stirring reactor (GRJB-5D, Zhenjiang Gerui Bioengineering Co., Ltd.) at 27°C, 150 rpm, for 7 days (Supplementary Materials and Methods S2).

### 2.2 Analytical methods

#### 2.2.1 Determination of intracellular and extracellular Cu content

With the optimal fermentation medium, *T. versicolor* was induced to produce laccase by GHK-Cu and the same amount of CuSO_4_ and the control with no inducer. During fermentation (12, 24, 48, 72, 96, 120, 144 h), 50 mL of the broth were collected and centrifugated at 10,000 rpm for 15 min. The supernatant and pelleted mycelium were collected. The pelleted mycelium was washed with sterile distilled water 3 times and vacuum dried. 100 mg of dry mycelium and 3 mL of nitric acid were added into the digestion tube for cold digestion for 1 h, and then, 5 mL water was added into the digestion tube for further digestion according to the following procedure. The digestion temperature rose to 130°C and was maintained for 20 min. After that, the temperature rose to 160°C and was maintained for 10 min, and then the temperature rose to 175°C and was maintained for 20 min. After cooling, constant volume of digestion liquid to 50 mL and used for the test of intracellular Cu content. A certain amount of the supernatant was also digested according to the above procedure and the resulted digestion liquid was used for the test of extracellular Cu content. The Cu content in digestion liquid was detected by ICP-MS. The content of extracellular Cu^2+^ was also determined by ICP-MS using the undigested supernatant directly.

GHK-Cu residues in fermentation were separated and analyzed using an Athena C^18^ -WP, 100A (150 mm × 4.6 mm, 5 µm) on a Shimadzu LC-10AT system equipped with an SPD-10AUV detector. The mobile phase was 0.1% trifluoroacetic acid (TFA) solution and methanol with an equal gradient of 95:5. The flow rate and injection volume were 0.6 mL min^−1^ and 20 μL, respectively, and the analytical wavelength was 220 nm.

#### 2.2.2 Microscopic observation of mycelium

As described in Section 2.2.1, the pelleted mycelia of *T. versicolor* were collected on the 7th day of fermentation and washed 3 times with pH 7, 0.2 mol L^−1^ PBS. Then, they were treated with 2.5% glutaraldehyde and 1% oxide for 2 h before being washed with 0.1 mol L^−1^ PBS. The rinsed samples were dehydrated overnight with 30% ethanol and rinsed with 0.1 mol L^−1^ PBS. Next, samples were transferred to a series of ethanol solutions (0, 70, 80, 90, and 100%) for 30 min, respectively. After dehydrating and embedding in Epon 812 resin, ultrathin sections were cut with a diamond knife. Then the slices were double-stained with uranyl acetate and lead citrate quickly. Finally, the slices were prepared and visualized by a transmission electron microscope (Hitachi H-7650, TEM). The experiments were carried out in triplicate. The thickness of the cell wall was measured near the section of the central part of the cell by Image J (V 1.8.0) software.

#### 2.2.3 Determination of laccase gene expression

As described in Section 2.2.1, the pellet mycelia of *T. versicolor* during fermentation process was collected (1, 2, 3, 4, 5, 6 and 7th day) and washed 3 times with sterile water, and the pelleted mycelium was dried with sterile cloth and stored at −80°C. RNA was extracted using TaKaRa MiniBEST Plant RNA Extraction Kit. gDNA removal and reverse transcription to cDNA synthesis was performed according to the Prime Script™ RT Reagent Kit with gDNA Eraser (Perfect Real Time). The synthesized cDNA was stored at −20°C. The primer sequences of 3 target genes laccase (*TvLac*) and housekeeping gene *18s* were standardized. The primers used in this study are shown in Supplementary Table S1. Real-time fluorescence quantitative PCR (RT-qPCR) was used in this study with the TB Green^®^ Premix Ex Taq™ II (Tli RNaseH Plus) Kit to perform the PCR reaction in the Step One Plus Real-time PCR System, and the reaction system was shown in Supplementary Table S2. The reaction was continued under the following conditions, 40 denaturation cycles at 95°C for 30 s, annealing, and extension steps at 60°C for 30 s. The melting curves ranged from 60°C to 95°C. The Bio-Rad CFX Manager 3.0 software was used to process the Ct (Threshold cycle) values and the 2^−ΔΔCT^ method was used to calculate the experimental results.

### 2.3 Other determination methods

Samples are taken daily from the fermentation broth, the culture broth was separated by centrifugation for 10 min at 8000 rpm, at 4°C. And then, cell-free supernatant was used to determine glucose content, extracellular protein content, and laccase activity. The glucose concentration was measured by an SBA-40E glucose tester. Extracellular protein was determined by Bradford. (1976), and bovine serum albumin was used as standard. The measurement of biomass and laccase activity was prepared according to the procedure described by Wang et al. (2014). In addition, to observe the leakage of intracellular substances, intracellular macromolecular leakage and conductivity were determined (Supplementary Materials and Methods S3).

### 2.4 Statistical analysis

All experiments were repeated three times, and results were analyzed using SPSS16.0 software (SPSS, Inc., Chicago, IL, United States) and expressed as mean ± standard deviation. The data were analyzed by one-way analysis of variance (ANOVA), and the difference between the means was tested by Tukey test (*p* < 0.05).

## 3 Results

### 3.1 Effects of GHK-Cu on laccase production

When the concentration of GHK-Cu was 50–200 μmol L^−1^, the production of laccase increased gradually, reaching the maximum value of 605.61 U L^−1^ at 200 μmol L^−1^. After that, laccase production remained stable with the increase of GHK-Cu concentration (Supplementary Figure S1). Therefore, GHK-Cu is an effective inducer of laccase of *T. versicolor*, and the expression of laccase was not affected by the high concentration of GHK-Cu, which may be due to its low cytotoxicity.

### 3.2 Screening of other important variables affecting laccase production

Seven variables were tested for the better laccase production by *T. versicolor* (Supplementary Figures S1, S2) using one-factor-at-a-time, GHK-Cu 200 μmol L^−1^, glucose 5 g L^−1^, NH_4_Cl 0.2 g L^−1^, KH_2_PO_4_ 0.2 g L^−1^, CaCl_2_ 0.04 g L^−1^, FeSO_4_·7H_2_O 0.03 g L^−1^, and MgSO_4_.7H_2_O 0.05 g L^−1^. According to the ANOVA, GHK-Cu, glucose, NH_4_Cl, KH_2_PO_4_, CaCl_2_, and FeSO_4_·7H_2_O were significant factors affecting laccase production with *p* < 0.05 (Supplementary Table S3). The production of laccase was improved and reached the maximal production at 200 μmol L^−1^ of GHK-Cu. Glucose of 10 g L^−1^ and NH_4_Cl of 0.2 g L^−1^ caused a maximum laccase production that was 635.48 and 533.27 U L^−1^, respectively. Different C/N is needed by different microorganisms, it leads to different glucose and NH_4_Cl contents in the culture medium. Laccase production was caused by a significant effect of KH_2_PO_4_, which plays an important role in cell growth and reproduction. The production of laccase reached 636.02 U L^−1^ with KH_2_PO_4_ of 0.2 g L^−1^. The effect of each parameter on laccase production was studied in the form of one-factor-at-a-time. However, the interaction between various factors is also very important in fermentation. Therefore, the composition of the fermentation medium was further optimized by BBD and RSM.

### 3.3 Optimization of screened variables by BBD-RSM

In a set of 54 trials, the optimal level of each component and their interactions were determined by BBD (Supplementary Table S4, S5). The quadratic model illustrated a mathematical relationship between the factors and laccase production as Supplementary Eq. SA6.

AVONA of the BBD was shown in Supplementary Table S4. A, B, C, AB, BF, and CE in the medium components were all significant. GHK-Cu, glucose, and NH_4_Cl were more influential than the other variables. In Supplementary Table S6, the F-value for “Lack of fit” was 2.51, indicating that the “Lack of fit” was not significant. The model had linear correlation coefficient (*R* ^2^) and the adjusted correlation coefficients (R^2^_adj_) were 0.95 and 0.91, respectively (Supplementary Tables S6, S7). A significant correlation between the predicted and the actual value of laccase production also was proved (Supplementary Figure S3). 3-D response surface (Supplementary Figure S4) was plotted through the optimal level of each variable and the effect of their interactions on laccase production. The interaction between glucose and GHK-Cu indicated that higher activity of laccase was observed at higher GHK-Cu concentration with increased glucose concentration until at the central level (Supplementary Figure S4A). The interaction between NH_4_Cl and KH_2_PO_4_ (Supplementary Figure S4B) showed that KH_2_PO_4_ and NH_4_Cl at the central level resulted in higher laccase production. As from Supplementary Figure S4C, D, the optimum laccase activity was obtained at high NH_4_Cl concentration with low concentration of CaCl_2_ and FeSO_4_·7H_2_O and this result is consistent with Supplementary Figure S4G. The response surface curve showed maximal laccase activity at the middle level of KH_2_PO_4_, CaCl_2_, and FeSO_4_·7H_2_O content (Supplementary Figures S4E, F).

Based on the above results, the formula was differentiated by software Design-Expert (Version 13, Stat-Ease Inc., United States), and the optimal medium formula was obtained as follows, GHK-Cu 290 μmol L^−1^, glucose 4.21 g L^−1^, NH_4_Cl 0.31 g L^−1^, KH_2_PO_4_ 0.18 g L^−1^, CaCl_2_ 0.047 g L^−1^, FeSO_4_.7H_2_O 0.043 g L^−1^, and MgSO_4_.7H_2_O 0.0526 g L^−1^. The maximum response value of the model is 830.233 U L^−1^.

To verify the induction of GHK-Cu on laccase production by *T. versicolor*, the laccase activity was 850.05 U L^−1^ (Supplementary Figure S5) at the 7th day of culture under optimal conditions, which was consistent with the predicted value. The production of laccase was 12.77-fold higher than that of the basal fermentation medium with the final enzyme activity of 66.59 U L^−1^. flask.

### 3.4 Laccase production in a 5 L reactor

**FIGURE 1:**
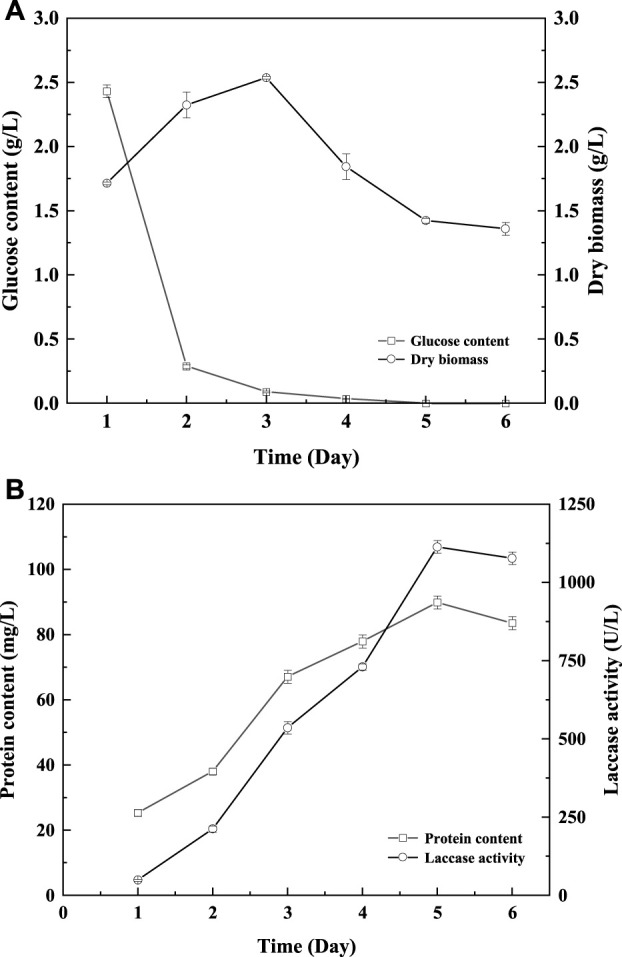
Changes of glucose content **(A)**, biomass **(A)**, protein content **(B)**, and laccase activity **(B)** during *Trametes versicolor* culture induced by GHK-Cu under optimal medium in reactor.

To further verify the feasibility of laccase production by fermentation induced by GHK-Cu, the scaling-up fermentation of *T. versicolor* in 5 L reactor was carried out. As shown in Figure 1, the growth trend of *T. versicolor* under amplification culture in the reactor is basically consistent with a shake flask (Supplementary Figure S5). In the first 72 h, *T. versicolor* grew rapidly, biomass reached the peak of 2.33 g L^−1^ on the 3rd day. At the same time, glucose was consumed rapidly until the 3rd day, which was consistent with the time when the maximum biomass of *T. versicolor* appeared. Laccase activity reached the maximum value of 1113.57 U L^−1^ on the 5th day. At the early stage of fermentation, with the cell growth and laccase production, the protein content was increased and reached the peak of 89.86 mg L^−1^ on the 5th day. Compared to the results in the shake flask, the peak of biomass and laccase production in a 5 L reactor was 1 day earlier than those in shake flask. And, the laccase production in bioreactor was 1.31-fold higher than that of the shake.

### 3.5 Possible mechanisms

#### 3.5.1 Effects of different inducer component and combination on laccase production

**FIGURE 2:**
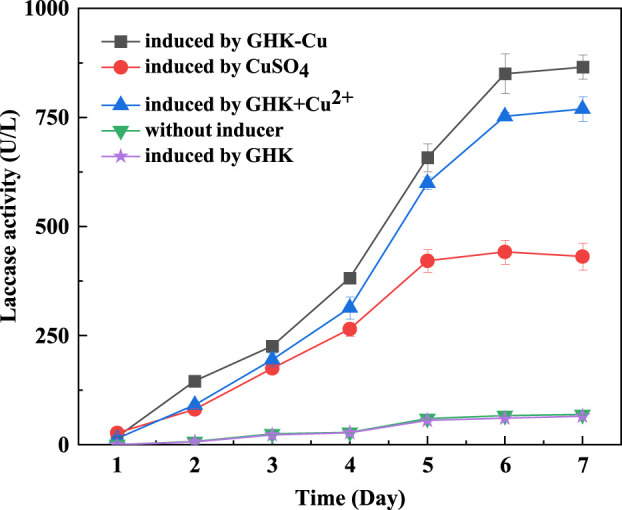
Effect of GHK-Cu, CuSO_4_, GHK+CuSO_4_, and GHK on laccase production.

Based on the optimal medium without GHK-Cu, the same amount of GHK-Cu, CuSO_4_, GHK+CuSO_4_, and GHK was added separately, and the control was without inducer. The laccase production of the five groups was compared and results are shown in Figure 2. It was can be seen that the addition of GHK-Cu, CuSO_4_ and GHK+CuSO_4_ could promote the laccase production by the *T. versicolor*, while GHK exhibited no promotion effect. Among them, GHK-Cu provided the highest laccase production of 850.05 U L^−1^, followed by GHK+CuSO_4_ complex solution with the laccase production of 752.52 U L^−1^, and CuSO_4_ exhibited the worst induction effect with the laccase production of 441.57 U L^−1^ on the 6th day. This suggested that GHK-Cu was the best inducer for laccase production by *T. versicolor* at the same concentration of Cu^2+^.

The effects of different concentrations of GHK-Cu and CuSO_4_ on the laccase production were also investigated and the results were shown in Supplementary Figure S6. Both GHK-Cu and CuSO_4_ could induce the laccase production when the concentration of Cu^2+^ was in the range of 50–300 μmol L^−1^. The laccase production reached the maximum of 850.05 U L^−1^ at 290 μmol L^−1^ GHK-Cu, while CuSO_4_ induced the peak laccase activity of 513.56 U L^−1^ at 400 μM. It indicated that the GHK-Cu exhibited high induction efficiency since higher laccase production was obtained at lower inducer concentration. In addition, further increase in inducer concentration resulted in no significant change in laccase production of the GHK-Cu group. However, the laccase production in the CuSO_4_ group significantly decreased at high concentration.

#### 3.5.2 Effect of GHK-Cu on membrane permeability

Supplementary Figure S7 showed the changes in leakage of protein and nucleic acid from the cells during the culture period of *T. versicolor*. The leakage of protein and nucleic acid from the same amount of cells increased with respect to the culture time. It indicated that the cell membrane permeability increased with the prolonged culture time. The treatment with CuSO_4_ or GHK-Cu could promote the cell membrane permeability of *T. versicolor* and CuSO_4_ showed the most serious destroy of cell membrane resulting in the largest amount of leakage. As shown in Supplementary Figure S8, the conductivity and mass transfer diffusion coefficient (R_c_) also increased with the increasement of culture time and CuSO_4_ showed the best performance.

**FIGURE 3:**
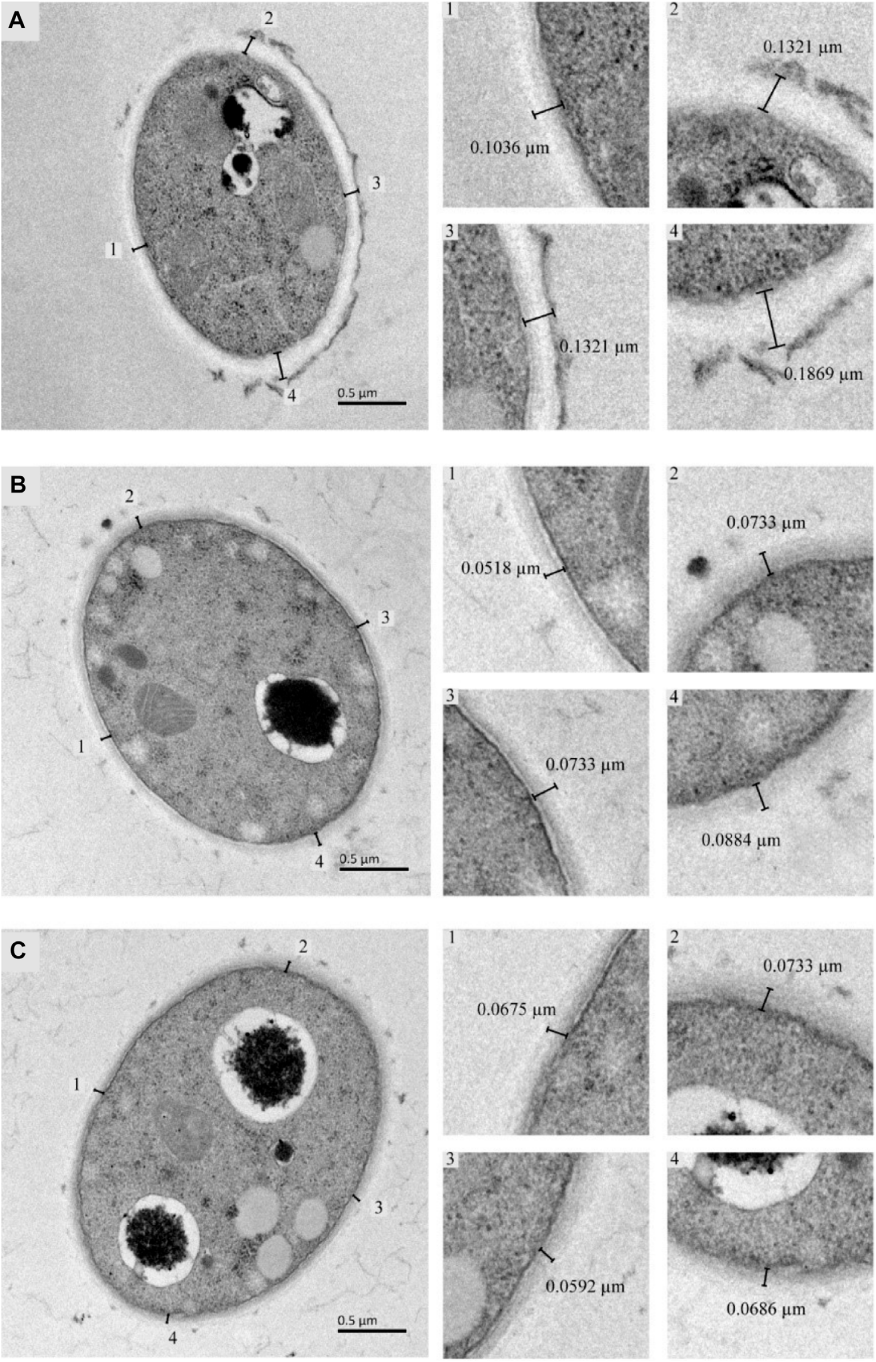
TEM analysis of *Trametes versicolor* cultured under different inducers [**(A)** Control, **(B)** GHK-Cu, **(C)** CuSO_4_].

The cell wall thickness of *T. versicolor* was observed by TEM (Figure 3). There was a significant difference in mycelial cell wall thickness. The control cell wall (0.139 ± 0.030 µm) was approximately 1.95-fold and 2.07-fold the thickness of the cells treated with GHK-Cu (0.071 ± 0.013 µm) and CuSO_4_ (0.067 ± 0.005 µm), respectively. This result proved that GHK-Cu and CuSO_4_ had an inhibitory effect on the cell wall synthesis of *T. versicolor* and CuSO_4_ exhibited more serious effect.

#### 3.5.3 Change of intracellular and extracellular Cu concentration

**FIGURE 4:**
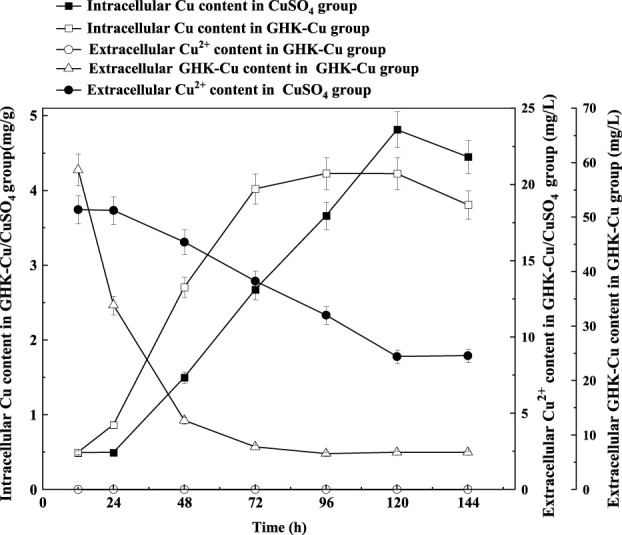
Changes of intracellular and extracellular copper content under different inducers.

**FIGURE 5:**
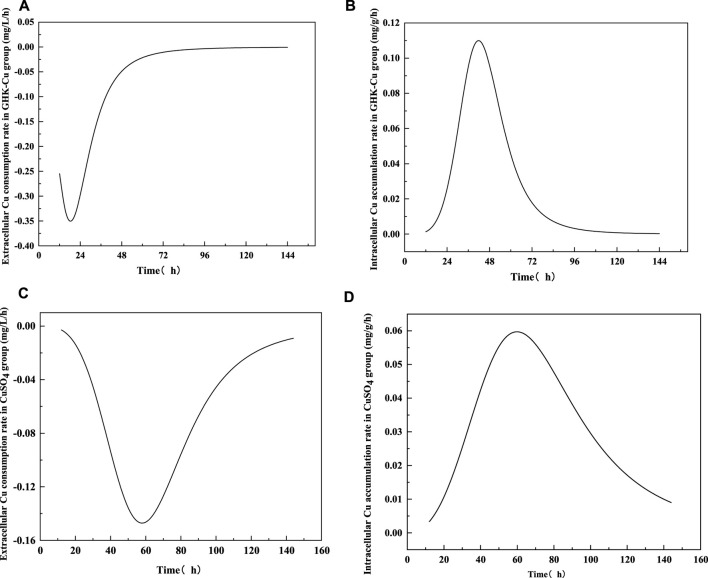
Consumption rates of extracellular Cu and accumulation rates of intracellular Cu treated with different inducers [**(A)** consumption rate in GHK-Cu group, **(B)** accumulation rate of Cu in GHK-Cu group, **(C)** consumption rate in CuSO_4_ group, **(D)** accumulation rate of Cu in CuSO_4_ group].

According to the results in Figure 4, no extracellular Cu^2+^ was detected in the GHK-Cu group, indicating that GHK-Cu in the broth was not disassociated or degraded into free Cu^2+^ during culture period. The consumption of extracellular GHK-Cu was faster, which tended to be stable after 72 h, and the consumption percentage reached 89.8% at 48 h, while that of Cu^2+^ in the CuSO_4_ group was only 12.7%. In this study, GHK-Cu, as a complex of small molecular protein and metal ions, facilitated the transportation of Cu into fungal cells in liquid culture. With the prolonged culture time, intracellular Cu showed a downward trend during 120–144 h in both treatment groups, which may be due to the leakage of intracellular Cu into the broth resulted from cell aging and autolysis. At 144 h, the mole concentration of the extracellular GHK-Cu in GHK-Cu group and the extracellular Cu^2+^ in CuSO_4_ group was 20 and 137 μmol L^−1^, respectively. However, the Cu content in mycelium was 3.73 and 4.45 mg g^−1^ for GHK-Cu group and CuSO_4_ group, respectively, where the biomass was 1.36 g L^−1^ in GHK-Cu group and 1.41 g L^−1^ in CuSO_4_ group (data not shown). These results indicated more Cu in the GHK-Cu group was consumed, which may be resulted from higher laccase production. The consumption rate of Cu and the accumulation rate of Cu in mycelium were also calculated (Figure 5). The maximal consumption rate and accumulation rate of Cu in the GHK-Cu group were 0.35 mg L^−1^ h^−1^ and 0.11 mg g^−1^ h^−1^ (Figures 5A, B). The peak of Cu consumption rate and Cu accumulation rate in CuSO_4_ group was 0.15 mg L^−1^ h^−1^ and 0.06 mg g^−1^ h^−1^ (Figures 5C, D). The maximal consumption and accumulation rates of Cu in GHK-Cu group were higher than those in the CuSO_4_ group and occurred in the earlier stage. The content of intracellular Cu in the GHK-Cu group also increased more rapidly than that in the CuSO_4_ group, and tended to be stable after 72 h.

#### 3.5.4 Analysis of laccase gene expression

**FIGURE 6:**
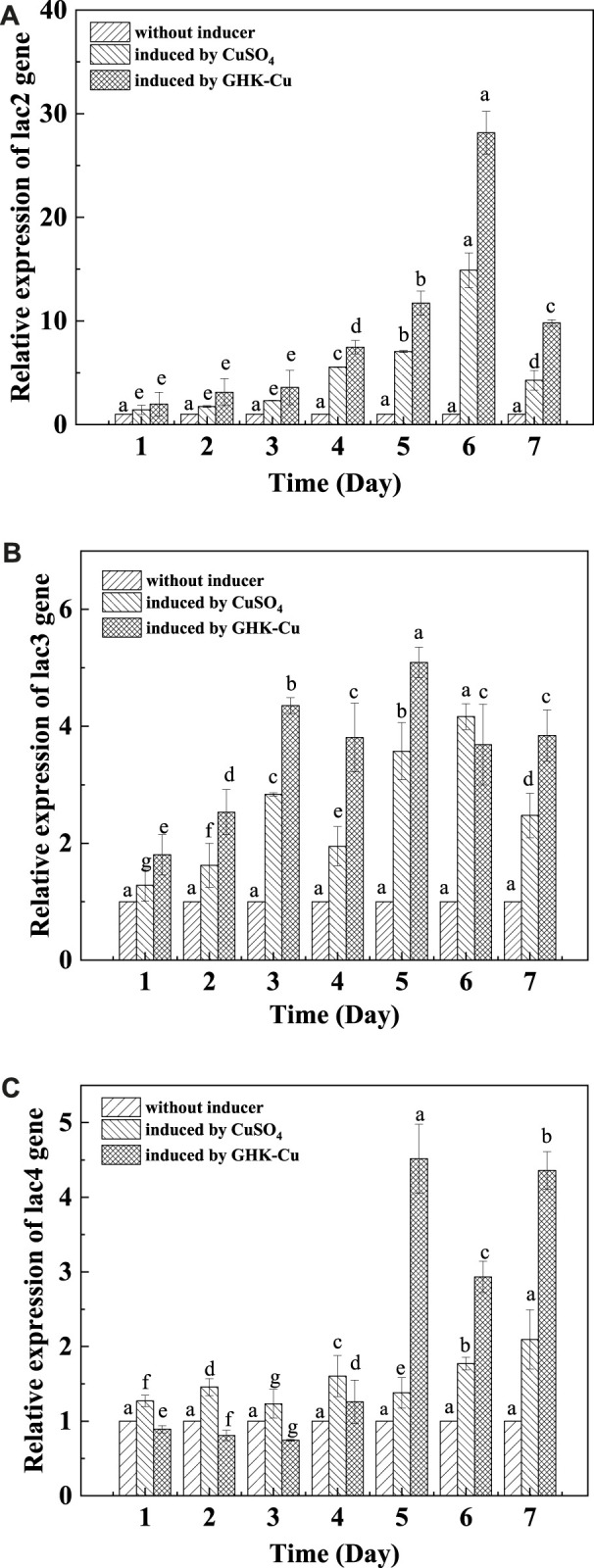
RT-qPCR analysis of laccase gene expression [**(A)** *TvLac2*, **(B)** *TvLac3*, **(C)** *TvLac4*].

The differential regulation of laccase gene expression (*TvLac2*, *TvLac*3 and *TvLac4*) during the fermentation period of *T. versicolor* was shown in Figure 6. The relative expression level of the tested laccase genes was relatively low in the early culture stage, and it increased gradually and reached the peak on the 5th and 6th day. The induced expression levels of *TvLac2* exhibited the highest relative increase multifold in both the GHK-Cu group and CuSO_4_ group, where it was 28.15 and 14.88 times higher than that of the control on the 6th day. The expression levels of *TvLac3* and *TvLac4* genes in the GHK-Cu group were 5.09 and 4.51 times than that of the control on the 5th day, respectively. The expression levels of *TvLac3* and *TvLac4* genes in the CuSO_4_ group were 4.16 and 2.09 times higher than those in the control on day 6 and day 7, respectively. Combined with Supplementary Figure S5, it was can be seen that the dynamic trend of gene expression level was basically the same as that of laccase production.

## 4 Discussion

### 4.1 Optimization of medium and scale-up of 5 L reactor for laccase production

In the exploration of the optimal composition of the culture medium, compared with other variables, GHK-Cu had a significant effect on laccase production. Cu^2+^ has been proved to be a good inducer of laccase production by *T. versicolor* (BİRhanli and Yesilada, 2017). This may be due to adding Cu^2+^ could upregulate the transcription level of the laccase gene (Xu et al., 2020). Metal ions have positive effects on laccase production (Akpinar and Ozturk Urek, 2017). In addition, inorganic salts play an important role in buffer and regulation between strain and culture medium, and are closely related to microbial growth and metabolism. Among them, laccase production was affected by KH_2_PO_4_, because it played an important role in the mass transfer of nutrients during microbial growth (Nandal et al., 2013). And, the same conclusion as Xu et al., was obtained, cell growth and metabolism were affected by C/N (Xu et al., 2020). Studies have shown that a higher concentration of glucose results in the faster growth of microbes and the better the products (Nandal et al., 2013). The results of this study are different from theirs. For different microorganisms, appropriate nitrogen source and carbon source both are important reason for increasing the laccase production (Wang et al., 2019; Su et al., 2020). Due to the different strains and media, the interaction between the various components of the medium is discrepant, so the optimal medium formulations to obtain the laccase production are diverse. Studies have proved that reasonable optimization of medium components can increase laccase production in fermentation (Patil et al., 2020). In 5 L reactor, the peak of biomass and laccase production was 1 day earlier than that in shake flask, and higher laccase production was obtained. The laccase production increased rapidly during the period of rapid glucose consumption. A similar phenomenon was also observed in the culture of *Aquatic Hyphomycetes*, which indicated a correlation between glucose consumption and biomass production (Charcosset and Chauvet, 2001). The better laccase production obtained in the bioreactor was due to the accurate control of temperature, pH, and effective supply of oxygen in the stirring reactor. Studies have shown that the supply and transfer of oxygen is an efficient strategy to promote the normal metabolism and product production of fungi (Meneghel et al., 2014).

### 4.2 Possible mechanisms

The effects of different inducers on laccase production were compared, and it was found that GHK-Cu had a better effect, which benefited from the characteristics of CHK-Cu. When GHK was coupled with copper, the peptide may quench the redox activity of copper, facilitating the non-toxic delivery of Cu^2+^ into the hepatoma cells (Pickart et al., 1980). This could be the reasons for the best laccase production induced by GHK-Cu. In case of GHK+CuSO_4_ addition, the complex GHK-Cu could be formed due to the high affinity GHK for Cu^2+^ (Alshammari and Platts, 2020). And thus, GHK+CuSO_4_ showed partly induction effect of GHK-Cu, resulting enhanced laccase production compared to that in CuSO_4_ group.

It can be found by analyzing the results of macromolecule leakage and Rc in the cells of *T. versicolor*. The increase of cell membrane permeability in CuSO_4_ group and GHK-Cu group was beneficial for the secretion of laccase, which can be one of the reasons for the enhanced laccase production. The same results can be obtained in TEM images, both GHK-Cu and CuSO_4_ could reduce cell wall thickness and enhance cell membrane permeability, while CuSO_4_ had a stronger effect. A significant decrease of cell wall’s thickness may be beneficial to the secretion of intracellular substance (Ma et al., 2019). However, the production of laccase induced by CuSO_4_ was lower, indicating that CuSO_4_ had a negative effect on laccase activity. These results proved that CuSO_4_ showed higher toxicity to *T. versicolor* cells than that of GHK-Cu. It has been reported that laccase production of *Ganoderma* sp. increased with the increasing Cu^2+^ concentration in the medium (Sharma et al., 2015) and high concentration of Cu^2+^ affected cell growth and laccase production of *Trametes* sp. due to its toxicity (Akpinar and Ozturk Urek, 2017). GHK-Cu showed no cytotoxicity to skin cells in the range of 0.0058–5,800 μmol L^−1^, while Cu^2+^ substantial cytotoxicity at 5,800 μmol L^−1^ after 30 min treatment (Li et al., 2016). It is maybe due to the promoted production of free radicals induced by excessive copper interfered with fatty acid and protein metabolism, respiration, and membrane integrity, resulting in the change of cell membrane permeability and the leakage of electrolyte (Shi-Sheng, 2007). It can be concluded that GHK-Cu was a good laccase inducer with high efficiency and low toxicity.

The content of copper inside and outside the cells of the suppository was determined. It indicated that GHK-Cu can enter cells quickly and be used efficiently compared to the Cu^2+^ from CuSO_4_. Lysine on the GHK side chain of GHK-Cu could participate in the recognition of liver cancer cell receptors, and these receptors played an important role in the absorption of Cu, the transport of Cu into cells was be promoted (Pickart et al., 1980). Therefore, the high-efficient absorption, accumulation and utilization of Cu could be one of the reasons for the good laccase expression induced by GHK-Cu. In fact, the promoter region of laccase gene may contain elements responding to Cu^2+^ regulation, which regulated laccase gene expression in a strain-dependent manner (Degerli et al., 2019; Pawlik et al., 2021). It has been proved that the increase in laccase gene expression level contributed to the increase in laccase production (Rodrigues et al., 2019). It can be concluded that GHK-Cu provided better expression level of laccase gene compared to CuSO_4_ at the same Cu^2+^ concentration, resulting in enhanced laccase production. It may be related to the quick and high accumulation of intracellular Cu.

All the above results indicate that GHK-Cu was more conducive to the secretion of laccase. This fact may be due to that GHK is considered to be the transporter of metal ions through membranes, and the exchange kinetics and REDOX behavior of GHK are stable in biological systems, which makes copper much safer for cells when transported into them (Alshammari and Platts, 2020). Moreover, glutathione chelating with Cu can be involved in repairing membrane damage caused by Cu (Freedman et al., 1899). It was speculated that GHK and glutathione have the same effect on reducing the damage of Cu to the cell membrane. Therefore, high production laccase was induced by GHK-Cu with less damage and toxicity to cells.

## 5 Conclusion

To obtain the efficient laccase production from *T. versicolor*, non-toxic GHK-Cu was used as a new inducer instead of CuSO_4_. The laccase production was greatly improved after the optimization of medium composition by one-factor-at-a-time, BBD and RSM, and it was scaled-up in 5 L stirring reactor. To reveal the possible mechanism, cell membrane permeability, copper consumption and accumulation and laccase gene expression were characterized. It was found that GHK-Cu was a low toxicity and high-efficient laccase inducer, which may expand the application of laccase in food industry by using the culture broth directly. Besides it, GHK chelated metal ions can also be a potential strategy for the induced expression of other metal enzymes.

## Data Availability

The original contributions presented in the study are included in the article/Supplementary Material, further inquiries can be directed to the corresponding author.
