# Supplementary material for: Improved laccase production by Trametes versicolor using Copper-Glycyl-L-Histidyl-L-Lysine as a novel and high-efficient inducer
Source: Front Bioeng Biotechnol. 2023 Apr 25;11:1176352. doi: 10.3389/fbioe.2023.1176352 (PMC10167017; doi:10.3389/fbioe.2023.1176352)
Supplement: Supplementary file 1 [file DataSheet1.docx]

Supplementary Material

Improved laccase production by *Trametes versicolor* using Copper-Glycyl-L-Histidyl-L-Lysine as a novel and high-efficient inducer

Feng Wang, Xiaolei Yu, Zhuo Yu, Yi Cui, Ling Xu^*^, Shuhao Huo, Zhongyang Ding, Liting Zhao, Lizhi Du and Yanguo Qiu

*** Correspondence:** Dr. Ling Xu; [lxu@ujs.edu.cn](mailto:lxu@ujs.edu.cn)

**Method and materials**

**1 Optimization of medium composition**

**1.1 Single factor optimization of basic culture components**

Various process parameters and GHK-Cu in *Trametes versicolor* fermentation were studied in this experiment. Effects of medium components on laccase production were tested by One-factor-at-a-time. The data were analyzed by Student’s t-test, and one-way ANOVA (Table S3) with Tukey’s multiple comparison test (*p*< 0.05).

**1.2 Box-Benhnken Design**

The Box-Behnken Design (BBD) experimental design in this study comprised of six significant factors, identified from the previous one-factor-at-a-time experiments, including glucose, NH_4_Cl, CaCl_2_, KH_2_PO_4_, FeSO_4_⋅7H_2_O, and MgSO_4_⋅7H_2_O. The variables were analyzed at three different levels (-1, 0, +1) and a set of 54 experiments was performed (Table S4&S5). The quadratic polynomial is obtained by fitting the experimental results with Design-expert, and the significance of the model is evaluated by analysis of variance (Table S5, S6). The behavior of the system was explained by the following quadratic equation (Eq. A1):

Y =Σβ_0_ + Σβ_i_ X_i_ +Xβ_i_ X_i_ X_j_ + Xβ_i_X_i_^2^ (Eq. A1),

where Y was the predicted response (laccase activity), β_0_ was the offset term, β_i_ was the linear coefficient, β_ii_ was the squared coefficient, β_ij_ was the interaction effect, and x_i_ was the dimensionless coded value of X_i_. The above equation was solved using the software Design-Expert (Version 7.0.2, Stat-Ease Inc., USA). And the interaction between medium compositions was analyzed by response surface methodology (RSM).

**2 Scaling-up of laccase production in 5 L reactor**

To evaluate the laccase production induced by GHK-Cu in a large scale, the cultivation of *T. versicolor* was conducted in a 5 L reactor under the optimized condition. 3 L of fermentation medium was added to a 5 L stirring reactor, and the defoaming agent was 1.5 mL soybean oil. The seed culture pellets of 300 mL were inoculated into the fermentation medium for laccase production. 300 mL 1.0 mol/L NaOH solution was connected to the fermenter system for pH control. The stirring reactor with fermentation medium and 1.0 mol L^-1^ NaOH solution was autoclaved for 20 min at 121 ^o^C before the inoculation. Sample and measure relevant data on time every day. All the scaling-up experiments were carried out in triplicate.

**3** **Determination of intracellular macromolecular leakage**

With the optimal fermentation medium, *T. versicolor* was induced to produce laccase by GHK-Cu and the same amount of CuSO_4_ and the control was no inducer. During fermentation (1, 2, 3, 4, 5, 6, and 7th day), 50 mL of the broth were collected and centrifugated at 10,000 rpm for 15 min. The same weight of mycelium was collected and washed with sterile distilled water 3 times in the three groups, and then it was suspended in sterile normal saline at 26 ^o^C and 150 rpm for 4 h. Finally, 5 mL suspension was centrifuged at 5000 rpm for 15 min, the supernatant was taken to determine the OD_260_ nm and OD_280_ nm values, and sterile normal saline was used as control.

The same method was used to collect the same weight of mycelium. And the mycelium was cleaned 3 times with 5% glucose isotonic solution (L_0_) and then re-suspended in the isotonic solution. The result showed that the conductivity of the final suspension (L_1_) was similar to that of 5% glucose solution. The suspension was shocked at 26^o^C and 150 r min^-1^ for 4 h, and the conductivity (L_2_) of the supernatant was measured by centrifugation. After that, the suspension was boiled in boiling water for 10 min. After cooling, the suspension was centrifuged to determine the conductivity L_0_'. Set three parallels for each group, and take the average value of the final result. The calculation formula was as follow (Eq. A2-5).

| Elative conductivity (%) = $\frac{(L2-L1)}{(L0^{'}-L0)}$ × 100% | (Eq. A2), |
| --- | --- |

where L_0_ (µS cm^-1^) was the conductivity of 5% glucose solution. L_0_' (µS cm^-1^) was the conductivity of the suspension after being boiled for 10 min after culture for 4 h. L_1_ (µS cm^-1^) was the initial conductivity of suspension. L_2_ (µS cm^-1^) was the conductivity of suspension culture after 4 h.

The process of electrolyte diffusion from the inside to the outside of the cell can be summarized by Fick's first law:

| -dC/dt=K_l_, sas (k-ke) | (Eq. A3), |
| --- | --- |

In other words, the membrane permeability Rc of *T. versicolor* can be expressed by mass transfer diffusion coefficient:

| Rc= K_l_ , sas= $\frac{d\kappa/dt}{k-ke}$ | (Eq. A4), |
| --- | --- |

The formula can be discretized to obtain:

| Rc (h^-1^) = $\frac{k_{1}-k_{0}}{(k_{2}-k_{0})\Delta t}$ | (Eq. A5), |
| --- | --- |

The measured value was Rc of the electrolyte diffusion coefficient of *T.versicolor* added with GHK-Cu, CuSO_4_, and blank control group.

where K_0_ (µS cm^-1^) was the conductivity of 5% glucose solution. K_1_ (µS cm^-1^) was the initial conductivity value of suspension. K_2_ (µS cm^-1^) was the conductivity of the suspension after being boiled for 10 min after 4 h culture. Δt (h) was the culture time of suspension.

**Results**

**1. The quadratic model of BBD-RSM**

$Y=751.18+75.62A-77.35B+157.62C+9.79D-14.05E-14.98F-74.02AB-22.31AC-14.46AD-11.03AE-33.19AF-31.42BC+36.55BD -9.15BE+43.18BF-3.02CD-81.72CE-25.74CF+11.56DE +2.63DF+35.14EF-59.67A^{2}-34.43B^{2}-223.95C^{2}-163.96D^{2}-85.93E^{2}-44.29F^{2}$(Eq. A6).

**Table Captions**

**Table S1** Laccase gene primer.

**Table S2** Reaction system of RT-qPCR.

**Table S3** ANOVA for one-factor-at-a-time design.

**Table S4** Experimental range and levels of independent variables studied by BBD in terms of actual and coded factors.

**Table S5** Experimental design and results of BBD of response surface methodology using 6 independent variables.

**Table S6** Analysis of variance of response surface quadratic model with 6 independent variables.

**Table S7** Fit statistics of response surface results.

**Figure Captions**

**Figure S1** Effect of different concentrations of GHK-Cu on laccase production.

**Figure S2** Effect of different medium compositions on laccase production ((A-F): Glucose, NH_4_Cl, KH_2_PO_4_, CaCl_2,_ FeSO_4_·7H_2_O, MgSO_4_·7H_2_O).

**Figure S3** Predicted and actual values for laccase production.

**Figure S4** 3-D surface and 2-D contour plots between glucose and GHK-Cu(A), NH_4_Cl and KH_2_PO_4_ (B), NH_4_Cl and CaCl_2_ (C), NH_4_Cl and FeSO_4_.7H_2_O (D), KH_2_PO_4_ and CaCl_2_ (E), KH_2_PO_4_ and FeSO_4_.7H_2_O (F), CaCl_2_ and FeSO_4_.7H_2_O (G) in laccase production.

**Figure S5** Changes in glucose content, biomass, protein, and laccase activity of extracellular during the culture of *T. versicolor* under optimal medium in shake flask.

**Figure S6** Effects of different concentrations of Cu^2+^ inducers on laccase production.

**Figure S7** Effects of different inducers on macromolecular leakage from *T. versicolor* ((A): nucleic acid, (B): protein).

**Figure S8** Influence of different inducers on the relative electrical conductivity of *T. versicolor* ((A): relative electrical conductivity, (B): mass transfer diffusion coefficient Rc).

**Wang et al., Table S1**

| laccase | gene ID | Upstream primer（5‘-3’） | Downstream primers（5‘-3’） |
| --- | --- | --- | --- |
| *18s* |  | GTAAAAGTCCTGGTTCCCC | CGATAACGAACGAGACCT |
| *TvLac2* | 19409505 | TTCAGCATCGACGACCACTC | GGTTTGCCTCCAACACGAAC |
| *TvLac3* | 19409052 | CCGCGGACCTCTCAGTTATC | ACCATCGATGCTGAACGTGT |
| *TvLac4* | 19407289 | CGTATGCGTCTTCTCTCGCT | GTCCGGCGTAGATCTGGATG |

**Wang et al., Table S2**

| Reagent | dosage（μL） |
| --- | --- |
| TB Green Premix Ex Taq II（Tli RNaseH Plus）（2X） | 10 |
| PCR Forward Primer（10 μM） | 0.8 |
| PCR Reverse Primer（10 μM） | 0.8 |
| ROX Reference Dye（50X） | 0.4 |
| DNA template | 2 |
| Sterile water | 6 |
| Total | 20 |

**Wang et al., Table S3**

| Element | Degree of freedom | F | P |
| --- | --- | --- | --- |
| glucose | 4 | 182.81 | ＜0.05 |
| NH_4_Cl | 4 | 84.79 | ＜0.05 |
| CaCl_2_ | 4 | 470.17 | ＜0.05 |
| KH_2_PO_4_ | 4 | 151.24 | ＜0.01 |
| FeSO_4_·7H_2_O | 4 | 104.77 | ＜0.05 |
| MgSO_4_·7H_2_O | 4 | 2.42 | 0.117＞0.05 |
| GHK-Cu | 4 | 204.28 | ＜0.05 |

**Wang et al., Table S4**

|  |  |  |  | Level of variable | | | |
| --- | --- | --- | --- | --- | --- | --- | --- |
| Element | Code value | Actual value | | | Code value | | |
|  |  | low | middle | high | low | middle | high |
| Glucose (g L^-1^) | A | 4 | 5 | 6 | -1 | 0 | 1 |
| NH_4_Cl (g L^-1^) | B | 0.1 | 0.3 | 0.5 | -1 | 0 | 1 |
| KH_2_PO_4_ (g L^-1^) | C | 0.1 | 0.2 | 0.3 | -1 | 0 | 1 |
| CaCl_2_ (g L^-1^) | D | 0.02 | 0.04 | 0.06 | -1 | 0 | 1 |
| FeSO_4_**^.^**7H_2_O (g L^-1^) | E | 0.01 | 0.03 | 0.05 | -1 | 0 | 1 |
| GHK-Cu (μM) | F | 100 | 200 | 300 | -1 | 0 | 1 |

**Wang et al., Table S5**

| Run | A | B | C | D | E | F | Actual value | (7d) Mean±SD |
| --- | --- | --- | --- | --- | --- | --- | --- | --- |
| 1 | 100 | 5 | 0.3 | 0.1 | 0.06 | 0.03 | 258.21 | 258.21±1.43 |
| 2 | 100 | 5 | 0.1 | 0.2 | 0.04 | 0.05 | 90.42 | 90.42±5.02 |
| 3 | 200 | 5 | 0.5 | 0.3 | 0.04 | 0.05 | 294.57 | 294.57±5.17 |
| 4 | 200 | 6 | 0.1 | 0.2 | 0.02 | 0.03 | 86.57 | 86.57±1.73 |
| 5 | 200 | 5 | 0.1 | 0.3 | 0.04 | 0.05 | 44.29 | 44.29±2.35 |
| 6 | 200 | 4 | 0.1 | 0.2 | 0.06 | 0.03 | 247.73 | 247.73±1.73 |
| 7 | 300 | 6 | 0.3 | 0.1 | 0.04 | 0.03 | 339.92 | 339.92±8.62 |
| 8 | 200 | 6 | 0.5 | 0.2 | 0.02 | 0.03 | 542.60 | 542.60±5.17 |
| 9 | 300 | 5 | 0.3 | 0.1 | 0.06 | 0.03 | 471.09 | 471.09±7.16 |
| 10 | 200 | 5 | 0.5 | 0.1 | 0.04 | 0.01 | 487.72 | 487.72±4.80 |
| 11 | 200 | 5 | 0.3 | 0.2 | 0.04 | 0.03 | 777.31 | 777.31±12.68 |
| 12 | 200 | 5 | 0.5 | 0.1 | 0.04 | 0.05 | 325.79 | 325.79±12.07 |
| 13 | 300 | 6 | 0.3 | 0.3 | 0.04 | 0.03 | 435.93 | 435.93±1.73 |
| 14 | 100 | 5 | 0.3 | 0.1 | 0.02 | 0.03 | 268.02 | 268.02±5.17 |
| 15 | 100 | 5 | 0.3 | 0.3 | 0.06 | 0.03 | 310.03 | 310.03±7.16 |
| 16 | 200 | 4 | 0.3 | 0.2 | 0.02 | 0.05 | 546.28 | 546.28±1.43 |
| 17 | 200 | 6 | 0.3 | 0.2 | 0.02 | 0.05 | 470.83 | 470.83±12.88 |
| 18 | 200 | 5 | 0.5 | 0.3 | 0.04 | 0.01 | 450.03 | 450.03±11.00 |
| 19 | 200 | 5 | 0.3 | 0.2 | 0.04 | 0.03 | 762.13 | 762.13±9.46 |
| 20 | 200 | 4 | 0.5 | 0.2 | 0.02 | 0.03 | 813.08 | 813.08±12.07 |
| 21 | 100 | 5 | 0.5 | 0.2 | 0.04 | 0.05 | 575.36 | 575.36±4.29 |
| 22 | 200 | 4 | 0.3 | 0.2 | 0.06 | 0.01 | 678.24 | 678.24±10.02 |
| 23 | 200 | 4 | 0.5 | 0.2 | 0.06 | 0.03 | 455.90 | 455.90±1.73 |
| 24 | 200 | 6 | 0.3 | 0.2 | 0.06 | 0.01 | 324.76 | 324.76±18.60 |
| 25 | 100 | 5 | 0.3 | 0.3 | 0.02 | 0.03 | 192.78 | 192.78±5.46 |
| 26 | 200 | 4 | 0.3 | 0.2 | 0.06 | 0.05 | 690.97 | 690.97±8.13 |
| 27 | 100 | 6 | 0.3 | 0.1 | 0.04 | 0.03 | 181.32 | 181.32±10.35 |
| 28 | 200 | 6 | 0.3 | 0.2 | 0.06 | 0.05 | 484.80 | 484.80±2.86 |
| 29 | 300 | 4 | 0.3 | 0.1 | 0.04 | 0.03 | 782.88 | 782.88±25.98 |
| 30 | 100 | 6 | 0.3 | 0.3 | 0.04 | 0.03 | 447.43 | 447.43±5.17 |
| 31 | 300 | 5 | 0.3 | 0.3 | 0.02 | 0.03 | 500.21 | 500.21±11.54 |
| 32 | 200 | 5 | 0.3 | 0.2 | 0.04 | 0.03 | 721.91 | 721.91±12.57 |
| 33 | 300 | 5 | 0.5 | 0.2 | 0.04 | 0.01 | 676.97 | 676.97±7.16 |
| 34 | 300 | 5 | 0.1 | 0.2 | 0.04 | 0.05 | 171.46 | 171.46±10.31 |
| 35 | 200 | 5 | 0.1 | 0.3 | 0.04 | 0.01 | 31.92 | 31.92±1.73 |
| 36 | 100 | 5 | 0.1 | 0.2 | 0.04 | 0.01 | 24.87 | 24.87±0.60 |
| 37 | 200 | 6 | 0.1 | 0.2 | 0.06 | 0.03 | 107.22 | 107.22±1.73 |
| 38 | 200 | 5 | 0.1 | 0.1 | 0.04 | 0.01 | 61.23 | 61.23±3.17 |
| 39 | 200 | 4 | 0.1 | 0.2 | 0.02 | 0.03 | 111.90 | 111.90±6.90 |
| 40 | 300 | 5 | 0.3 | 0.1 | 0.02 | 0.03 | 465.19 | 465.19±18.58 |
| 41 | 200 | 6 | 0.3 | 0.2 | 0.02 | 0.01 | 418.49 | 418.49±17.50 |
| 42 | 100 | 5 | 0.5 | 0.2 | 0.04 | 0.01 | 542.31 | 542.31±18.77 |
| 43 | 300 | 5 | 0.3 | 0.3 | 0.06 | 0.03 | 493.02 | 493.02±2.86 |
| 44 | 300 | 4 | 0.3 | 0.3 | 0.04 | 0.03 | 686.21 | 686.21±8.81 |
| 45 | 100 | 4 | 0.3 | 0.1 | 0.04 | 0.03 | 246.91 | 246.91±17.25 |
| 46 | 200 | 5 | 0.3 | 0.2 | 0.04 | 0.03 | 791.35 | 791.35±15.41 |
| 47 | 300 | 5 | 0.1 | 0.2 | 0.04 | 0.01 | 209.63 | 209.63±11.45 |
| 48 | 200 | 5 | 0.1 | 0.1 | 0.04 | 0.05 | 54.10 | 54.10±1.73 |
| 49 | 200 | 5 | 0.3 | 0.2 | 0.04 | 0.03 | 665.45 | 665.45±17.33 |
| 50 | 200 | 5 | 0.3 | 0.2 | 0.04 | 0.03 | 788.91 | 788.91±21.06 |
| 51 | 300 | 5 | 0.5 | 0.2 | 0.04 | 0.05 | 486.60 | 486.60±19.46 |
| 52 | 200 | 4 | 0.3 | 0.2 | 0.02 | 0.01 | 772.19 | 772.19±17.51 |
| 53 | 100 | 4 | 0.3 | 0.3 | 0.04 | 0.03 | 345.51 | 345.51±17.25 |
| 54 | 200 | 6 | 0.5 | 0.2 | 0.06 | 0.03 | 250.82 | 250.82±5.15 |

**Wang et al., Table S6**

| Source | Sum of squares | Degrees of freedom | Mean square | F | P |  |
| --- | --- | --- | --- | --- | --- | --- |
| Model | 1.881E+06 | 27 | 69683.17 | 19.76 | < 0.0001 | significant |
| A-GHK-Cu | 1.372E+05 | 1 | 1.372E+05 | 38.91 | < 0.0001 |  |
| B-Glucose | 1.436E+05 | 1 | 1.436E+05 | 40.71 | < 0.0001 |  |
| C-NH_4_Cl | 5.962E+05 | 1 | 5.962E+05 | 169.03 | < 0.0001 |  |
| D-KH_2_PO_4_ | 2301.50 | 1 | 2301.50 | 0.6525 | 0.4266 |  |
| E-CaCl_2_ | 4735.47 | 1 | 4735.47 | 1.34 | 0.2571 |  |
| F-FeSO_4_**^.^**7H_2_O | 5384.26 | 1 | 5384.26 | 1.53 | 0.2277 |  |
| AB | 43835.38 | 1 | 43835.38 | 12.43 | 0.0016 |  |
| AC | 3981.86 | 1 | 3981.86 | 1.13 | 0.2978 |  |
| AD | 3345.15 | 1 | 3345.15 | 0.9484 | 0.3391 |  |
| AE | 973.95 | 1 | 973.95 | 0.2761 | 0.6037 |  |
| AF | 8813.89 | 1 | 8813.89 | 2.50 | 0.1260 |  |
| BC | 7898.93 | 1 | 7898.93 | 2.24 | 0.1466 |  |
| BD | 10685.83 | 1 | 10685.83 | 3.03 | 0.0936 |  |
| BE | 1338.58 | 1 | 1338.58 | 0.3795 | 0.5432 |  |
| BF | 14913.42 | 1 | 14913.42 | 4.23 | 0.0499 |  |
| CD | 73.00 | 1 | 73.00 | 0.0207 | 0.8867 |  |
| CE | 53426.38 | 1 | 53426.38 | 15.15 | 0.0006 |  |
| CF | 10599.73 | 1 | 10599.73 | 3.01 | 0.0949 |  |
| DE | 1069.72 | 1 | 1069.72 | 0.3033 | 0.5865 |  |
| DF | 55.46 | 1 | 55.46 | 0.0157 | 0.9012 |  |
| EF | 9878.77 | 1 | 9878.77 | 2.80 | 0.1062 |  |
| A² | 36605.23 | 1 | 36605.23 | 10.38 | 0.0034 |  |
| B² | 12194.94 | 1 | 12194.94 | 3.46 | 0.0743 |  |
| C² | 5.159E+05 | 1 | 5.159E+05 | 146.25 | < 0.0001 |  |
| D² | 2.765E+05 | 1 | 2.765E+05 | 78.39 | < 0.0001 |  |
| E² | 75953.23 | 1 | 75953.23 | 21.53 | < 0.0001 |  |
| F² | 20177.21 | 1 | 20177.21 | 5.72 | 0.0243 |  |
| Residual | 91710.47 | 26 | 3527.33 |  |  |  |
| Lack of fit | 83774.16 | 21 | 3989.25 | 2.51 | 0.1553 | not significant |
| Pure error | 7936.31 | 5 | 1587.26 |  |  |  |
| [Total](javascript:;) | 1.973E+06 | 53 |  |  |  |  |

**Wang et al., Table S7**

| Std. Dev. | 59.39 | R² | 0.9535 |
| --- | --- | --- | --- |
| Mean | 337.63 | Adjusted R² | 0.9053 |
| C.V. % | 17.59 | Predicted R² | 0.7724 |
|  |  | Adeq Precision | 14.9249 |

**Wang et al., Figure S1**

**Wang et al., Figure S2**

|  |  |
| --- | --- |
|  |  |
|  |  |

**Wang et al., Figure S3**


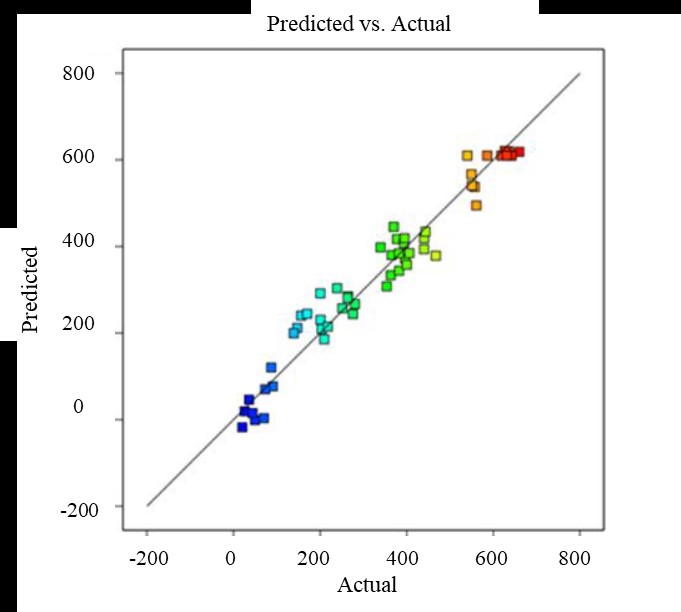


**Wang et al., Figure S4**

-
-
-

**Wang et al., Figure S5**

|  |
| --- |
|  |

**Wang et al., Figure S6**

**Wang et al., Figure S7**

**Wang et al., Figure S8**
